# Shifts at The Helm: gratitude, re-commitment to our work, and a call for addictions disparities research

**DOI:** 10.1186/s13722-022-00290-w

**Published:** 2022-02-18

**Authors:** Emily C. Williams, Jeffrey H. Samet

**Affiliations:** 1 Department of Health Systems and Population Health, University of Washington School of Public Health, Seattle, WA USA; 2 Clinical Addiction Research and Education (CARE) Unit, Section of General Internal Medicine, Department of Medicine, Boston University, School of Medicine and Boston Medical Center, Boston, MA USA

In 2011, *Addiction Science & Clinical Practice* (*ASCP*) Editor-in-Chief Jeffrey Samet (with the late Editor Emeritus Richard Saitz) took the reins of our journal from the National Institute on Drug Abuse to usher it into a new era as an open-access forum for clinically relevant research and perspectives that contribute to improving the quality of care (prevention and treatment) for people with unhealthy alcohol, tobacco, or other drug use and addictive behaviors across a spectrum of settings. Now, as we begin our 11th year, we can look back at our accomplishments over the last decade: we have published 766 articles (44% from outside the US, 26% from Europe); produced 5 thematic series, 1 special issue, and 10 supplements; and forged lasting partnerships with the International Network on Brief Interventions for Alcohol and Drugs (INEBRIA) and the Addictions Health Services Research (AHSR) Conference. *ASCP* has been cited 7277 times with a yearly average 12% increase that jumped to 28% in the last year. Notably in 2020, we received a first-time Journal Impact Factor of 3.088, 7th of 20 addiction journals (it has since climbed to 3.544 in 2021). We are proud of these accomplishments and grateful to our team of outstanding associate editors, our editorial board and managing editor, our contributors, and all of you who contribute your time to review articles for us.

The work of our *ASCP* community has never been more relevant. Though addiction is a consistent and ubiquitous threat to public health across cultures and societies globally, roughly over the same period as our journal was gaining momentum, substance use reached an all-time high. The US opioid overdose crisis hit peak levels of deaths and increasingly became characterized by polysubstance use with particularly large increases in methamphetamine use among people who use opioids [1]. Overdose deaths from cocaine and methamphetamine increased 3- and 13-fold, respectively [2, 3]. Unhealthy alcohol use increased substantially, particularly in women; alcohol-related deaths doubled between 1999 and 2017 and served as a major contributor to the decline in overall US life expectancy observed between 2015 and 2017 [4]. Though tobacco use has declined overall worldwide, use has increased among men; 45% of adults in South East Asia use tobacco [5], and vaping among youth increased ~ 75% between 2017 and 2018 with greater increases expected annually [6]. Finally, both medical and recreational cannabis use have become increasingly legal and accessible, creating a need for studies about its epidemiology and treatment, particularly its longitudinal health impacts [7]. This time was also marked by new funding mechanisms and multiple innovative and rigorous efforts to implement evidence-based care for substance use in medical settings [8–11] and innovative clinical trials [12–14] the results of which have informed clinical guidelines [15] and ongoing conversations about best practices [16–18]. Despite these innovations, the majority of persons with unhealthy substance use, substance use disorders, and/or other addictive disorders do not receive evidence-based care [19, 20].

Likely stemming from structural fundamental causes, adverse social and health consequences from substance use and barriers to care disproportionately impact Black, Indigenous, and other Persons of Color (BIPOC); persons with minoritized sexual and gender identities; persons living with HIV and Hepatitis-C; and other groups with lived experiences that increase risk for lower access to resources [21–25]. Syndemic public health crises—structural racism and the novel Coronavirus (COVID-19)—have together and exacerbated and highlighted the needed work toward treating and preventing substance use disorders. Structural racism, or historically-rooted and culturally reinforced discrimination through mutually reinforcing systems (e.g., in housing, education, employment, credit, income, and criminal justice), shapes access to resources and has specifically fundamentally shaped responses to substance use. This is particularly true in the US, where structural racism has influenced addictions stigma and treatment through differential enforcement of substance use policy (disproportionate arrest, incarceration, and mandatory substance use treatment of BIPOC folks for drug-related charges), greater stigma and criminalization of substance use when it occurs in non-White communities, and racially-patterned substance regulation and discrimination [26]. The introduction of the COVID-19 pandemic has generated a “perfect storm” with regard to substance use—both consequences and its treatment, particularly for BIPOC and individuals from other marginalized groups. Existing research on COVID-19 suggests that living in the context of a global pandemic with related healthcare and social restrictions has been associated with increased use and severity of substances [27], increased overdose deaths [28, 29], and less access to syringe service programs and other community treatment resources that serve large populations of those most affected by structural racism and its sequelae [30]. Tangential but related to addictions, COVID-19 has also contributed to peak levels of comorbid mental health disorders [31], disproportionately killed BIPOC populations, and compounded pre-existing health disparities along racial lines related to systematic societal disadvantage [32]. Changes to the healthcare system resulting from the pandemic (including those focused specifically on addictions health services, such as federal relaxation of buprenorphine induction standards) hold potential to permanently and dramatically reshape US addiction healthcare. The consequences of such policy changes are unknown—they could ameliorate or exacerbate existing health and healthcare disparities in addictions [33, 34].

In short, our addiction field is ripe for new and innovative studies focused on understanding disparities and their structural origins; development of new and innovative clinical practices to address old and emerging challenges in addictions treatment; and rigorous evaluations of policies ushered in as a result of the global pandemic that has disproportionately influenced vulnerable and historically stigmatized populations.

*ASCP* is excited to usher in this new era of high-quality addictions disparities research. To help steward that work, we are delighted to introduce our new co-Editor-in-Chief, Emily Williams, PhD, MPH. Dr. Williams is a mixed-methods implementation scientist and disparities researcher whose portfolio focuses on increasing access to evidence-based treatment and prevention for unhealthy alcohol and opioid use in medical and community-settings. She is a professor at the University of Washington School of Public Health within the Department of Health Services and has been serving as an Associate Editor of *ASCP* since Drs. Samet and Saitz assumed editorial control of the journal in 2011. Defining, understanding, and addressing disparities in addictions outcomes and treatments is a substantial focus of her work.

To promote rigorous addiction health services disparities work that focuses on structural racism, white supremacy, and stigma as fundamental causes of inequalities in substance use and its treatment [35, 36], we are strongly encouraging new submissions of manuscripts reporting clinically-relevant epidemiologic, intervention, and evaluation research that focuses on the broader social forces that shape disparities in the treatment or prevention of substance use disorders. We aim to increase our publication of research focused on structures (e.g., racism or gender discrimination) instead of minoritized identities or individual characteristics (e.g., race or gender) [36] and research that draws on sound theory (e.g., Critical Race Theory [37] and the Public Health Critical Race Praxis [38, 39]) and recommended equity approaches to knowledge production (e.g., community-based participatory research) [40]. Though *ASCP*’s decisions to publish manuscripts are solely based on research and not the researchers, we strongly support diversification of the field of addictions researchers and encourage submissions from diverse investigators in and out of the US [41].

We are excited to move this work forward together, and to be recommitting to providing a top-notch forum for clinically relevant research and perspectives that contribute to improving both the quality and equity of addictions care.
